# The incidences of acute mesenteric ischaemia vary greatly depending on the population and diagnostic activity

**DOI:** 10.1186/s13054-024-04870-x

**Published:** 2024-03-18

**Authors:** Annika Reintam Blaser, Kadri Tamme, Joel Starkopf, Alastair Forbes, Marko Murruste, Peep Talving, Stefan Acosta, Martin Björck

**Affiliations:** 1 Institute of Clinical Medicine, University of Tartu, Tartu, Estonia; 2 Department of Intensive Care Medicine, Lucerne Cantonal Hospital, Lucerne, Switzerland; 3 Tartu University Hospital, Tartu, Estonia; 4 North Estonia Medical Centre, Tallinn, Estonia; 5 Department of Clinical Sciences, Lund University, Malmö, Sweden; 6 Department of Surgical Sciences, Vascular Surgery, Uppsala University, Uppsala, Sweden

We much appreciate the interest of Drs Gazelli and Nacher regarding the AMESI study [1, 2], and for their effort to debate the difficulties in establishing a “true incidence” of acute mesenteric ischaemia (AMI) [3]. To address the question of true incidence, we first need to acknowledge the multifaceted nature of AMI. The main drivers of arterial occlusive AMI are cardiac arrhythmias (that increase exponentially with age) explaining most embolic occlusions, and smoking, which is the most important risk factor for thrombotic occlusion [4–6]. The non-occlusive arterial AMI (NOMI) is mainly associated with intensive care practices, as well as the incidences of sepsis and heart surgery [1, 6]. The main risk factors for venous AMI are obesity, previous venous thromboembolism and genetic thrombophilia [1, 6, 7]. Given this complex pathophysiological background, it is not surprising that the crude incidence rates vary depending on the studied population [8]. Those risk factors, as well as demography, likely vary greatly between regions, countries and hospitals across the world. The estimated incidence of AMI and its subtypes is unknown in most countries, except in Estonia, Sweden, and Finland, where population-based studies have recently been conducted, the latter two also declaring autopsy rates in their respective populations [9–11]. As the authors rightly imply, we lack the detailed knowledge on the incidence of the different entities of AMI in low–middle-income countries. A parallel may be drawn with the cardiovascular disease with largely variable incidences and trends between countries, where the burden in high-income countries may decline, while increasing in low–middle-income countries [12, 13], perhaps with increasing incidence of AMI.

Considering these diverging risk factors, which result in different incidences of AMI, there are likely relevant differences in the capabilities of healthcare systems to identify and treat AMI. The awareness of AMI may vary, and it is likely that a number of AMI cases went undetected during the AMESI study. However, this also mirrors the true current situation, as the detected cases were included in the study. Sites having an intensivist as a principal investigator were possibly more likely to identify patients with NOMI. Interestingly, the only specialist centre in the AMESI study did not identify any patients with NOMI, despite having the highest rate of other subtypes of AMI included. The main diagnostic modality, a true game-changer during the recent decades, is the tri-phasic computed tomography angiography (CTA) [6]. This is a rather expensive diagnostic modality, and it is not readily available in all low–middle-income countries, especially not in rural areas. The frequency of post-mortem examinations is low in all countries, in fact making it impossible to identify the true incidence of AMI, because of inability to identify AMI as an undetected cause of death among those not diagnosed alive.

When planning the AMESI study, we made an effort to include different types of hospitals, from peripheral district hospitals to one national referral centre for intestinal ischaemia. The 32 hospitals are located in three continents: Asia, South America and Europe. This variability will affect the individual incidences, but also add to the generalizability of the conclusions. The fact that the AMESI is by far the largest prospective study ever performed on AMI does make it possible to perform several subgroup analyses. Multiple such are underway, we have only published our first analysis [1]. However, regarding the true incidence of AMI, the AMESI study is probably just as good as it gets in hospital settings.

## Data Availability

Not applicable.
